# Ferulic acid (FA) protects human retinal pigment epithelial cells from H_2_O_2_‐induced oxidative injuries

**DOI:** 10.1111/jcmm.15970

**Published:** 2020-10-20

**Authors:** Kunpeng Xie, Bo Jin, Haiyan Zhu, Pengyi Zhou, Liping Du, Xuemin Jin

**Affiliations:** ^1^ Department of Ophthalmology the First Affiliated Hospital of Zhengzhou University Henan Province Eye Hospital Henan International Joint Research Laboratory for Ocular Immunology and Retinal Injury Repair Zhengzhou China

**Keywords:** age‐related macular degeneration, apoptosis, ferulic acid, hydrogen peroxide, oxidative stress

## Abstract

The aim of present study is to investigate whether Ferulic acid (FA), a natural polyphenol antioxidant, was able to protect ARPE‑19 cells from hydrogen peroxide (H_2_O_2_)‑induced damage, and elucidate the underlying mechanisms. Our results revealed that FA pre‐treatment for 24 hours can reverse cell loss of H_2_O_2_‐induced ARPE‐19 cells via the promotion of cell proliferation and prevention of apoptosis, as evidenced by 5‐ethynyl‐2′‐deoxyuridine (EdU) incorporation and terminal deoxynucleotidyl transferase‐mediated dUTP nick end‐labelling (TUNEL) assay, respectively. Moreover, the addition of FA (5 mM) can decrease Bax and cleaved caspase‐3 protein expression, but increase Bcl‐2 protein expression in ARPE‐19 cells. Furthermore, H_2_O_2_‐induced oxidative stress in ARPE‐19 cells was significantly alleviated by FA, illustrated by reduced levels of ROS and MDA. In addition, the attenuated antioxidant enzymes activities of (SOD, CAT and GPX) and GSH level were reversed almost to the normal base level by the pre‐addition of FA for 24 hours. In all assays, FA itself did not exert any effect on the change of the above parameters. These novel findings indicated that FA effectively protected human ARPE‐19 cells from H_2_O_2_‐induced oxidative damage through its pro‐proliferation, anti‐apoptosis and antioxidant activity, suggesting that FA has a therapeutic potential in the prevention and treatment of AMD.

## 1 INTRODUCTION

Age‑related macular degeneration (AMD) is one of the top causes of irreversible visual injury in the elderly throughout the world and epidemiological reports have noted that the number of aged patients with AMD is increasing steadily every year.^1^, ^2^ Despite affecting millions of people, current effective treatments available for this disease are still limited and restricted to its complex pathogenesis.^3^ Although the exact mechanisms of AMD formation remain largely elusive, multiple factors such as gene, environment, behaviour and nutrition are attributed to the pathogenesis of AMD.^4^, ^5^ More specifically, growing evidence has revealed that oxidative stress‐induced dysfunction of retinal pigment epithelium (RPE) cells leading to secondary photoreceptor loss serves as the most powerful pathological cause in the early aetiology of AMD.^6^, ^7^ Thus, preventing oxidative stress‐induced RPE cell death is the only strategy accessible to prevent or delay AMD progression in early stage.

Recently, consumer preference for naturally occurring antioxidant agents (including polyphenols, flavonoids, carotenoids, vitamins and minerals), which are found to be effective in reducing the risk of developing advanced AMD, has been rising.^5^, ^8^, ^9^ In particular, polyphenols are excellent antioxidant agent, which are abundant in plants, vegetables and fruits. Increasing evidence has suggested that some polyphenol antioxidants such as quercetin,^9^ resveratrol,^10^ blueberry anthocyanins^8^, ^9^ and epigallocatechin‐3‐gallate,^11^ can afford protective effects against cell death induced by oxidative stress in RPE cells.

Ferulic acid (FA) is of particular interest as a potent natural antioxidant, which has the ability to scavenge free radicals and prevent lipid peroxidation^12^; therefore, it has been proposed as a potential therapeutic agent for treating many radical‐induced disease including cardiovascular diseases, cancer, diabetes mellitus and Alzheimer's disease (AD).^13^ Recently, FA was reported to exert potent protective effect on retinal damage induced by oxidative stress in vitro and in vivo,^14^, ^15^ indicating it has potential in delaying or preventing the progression of AMD. However, the exact anti‐oxidative effects of FA on the ageing RPE cells are still unclear, let alone the underlying molecular mechanism. In this regard, the purpose of this study was to investigate the effects of FA on RPE cells challenged by H_2_O_2_ in detail and gain further insight into the mechanism involved in this effect.

## 2 MATERIALS AND METHODS

### 2.1 Materials and chemicals

FA (purity > 99.5%), 3‐(4,5‐Dimethylthiazol‐2‐yl)‐2,5‐diphenyltetrazolium bromide (MTT) and DMSO were obtained from Sigma (St. Louis, MO, USA). Dulbecco's modified Eagle medium (DMEM) and foetal bovine serum (FBS) were purchased from Invitrogen Co. (Grand Island, NY, USA). Primary antibodies against Bax, Bcl‐2, cleaved caspase‐3 and β‑actin antibodies were obtained from Santa Cruz Biotechnology, Inc (Santa Cruz, CA, USA). Superoxide dismutase (SOD), Catalase (CAT), GSH‐peroxidase (GPX), malondialdehyde (MDA), glutathione (GSH), lactic dehydrogenase (LDH) and terminal deoxynucleotidyl transferase‐mediated dUTP nick end‐labelling (TUNEL) Death Detection kits were from Nanjing Jiancheng Bioengineering Institute (Nanjing, Jiangsu, China). Horseradish peroxidase‐conjugated secondary antibody and enhanced chemiluminescence (ECL) reagent were obtained from Pierce (Rockford, IL, USA). All other chemical reagents used in this experiment were of analytical grade.

### 2.2 Cell culture and treatment

The human ARPE‐19 cells were purchased from the American Type Culture Collection (Manassas, VA, USA) and maintained in DMEM/F‐12 supplemented with 10% (v/v) FBS, 100 U/mL penicillin, 100 μg/mL streptomycin and 4 mM L‐glutamine in a humidified incubator at 37°C in the presence of 5% CO_2_. The culture medium was replaced every 2‐3 days and these cells of passages 5 were used in further experiments. ARPE‐19 cells (5 × 10^4^ cells/well) were seeded into 96‐well plates and cultured to confluence overnight. The ARPE‐19 cells were treated with different concentrations of H_2_O_2_ (0‐0.5 mM) for 4h to determine the working concentration of H_2_O_2_. The ARPE‐19 cells were treated with different concentrations of FA (0, 0.25, 0.5, 1, 2, 5, 10 mM) for 24 hours to determine the optimal concentration for FA. The ARPE‐19 cells were pre‐treated with FA at designated concentration for 24 hours before exposure to H_2_O_2_ (300 μM) for 4 hours to determine the protective effect of FA on H_2_O_2_‐induced cell loss. The concentrations of H_2_O_2_ and FA, as well as the incubation periods, were preliminarily determined in the pilot studies.

### 2.3 MTT assay

The MTT assay was used to investigate cell viability. Briefly, after different treatment, cells were added with 200 μL medium containing 20 μL of MTT (5 mg/mL) and incubated overnight at 37°C for 4 hours. Then, the medium was aspirated and 150 μL of DMSO was added to each well to dissolve formazan crystals with gentle agitation. The absorbance was measured at a wavelength of 490 nm using a microplate reader (BioTek, Winooski, VT, USA). Cell viability (%) was calculated as follows: [(mean absorbance of the sample‐reference absorbance)/ mean absorbance of the control] ×100.

### 2.4 LDH assay

ARPE‐19 cells seeded in 96‐well microplates at a density of 5 × 10^4^ cells/well were treated FA for 24 hours, and then allowed to exposure with H_2_O_2_ (300 μM) for another 4 hours. Afterwards, aliquots of the conditioned cell supernate were isolated for LDH measurement using a commercial LDH kit according referring to manufacturer's instructions. The results were expressed as the percentage of the control (100%).

### 2.5 5‐ethynyl‐2′‐deoxyuridine (EdU) incorporation assay

After treatment with FA as describe above in six‑well tissue culture plates, cells rinsed three times with cold PBS and fixed with 4% formaldehyde in PBS for 20 minutes at room temperature. Then, the cells were washed three times with cold PBS and stained with an EdU labelling/detection kit in compliance with the manufacturer's instructions. After staining, the cells were further washed thrice with PBS and photographed under a fluorescence microscope (Agilent 1200; Agilent Technologies).

### 2.6 TUNEL assay

Apoptotic cell death was measured using a commercially available TUNEL assay kit following the manufacturer's protocols. Briefly, cells with different treatment on coverslips were washed with cold PBS buffer and fixed with freshly prepared 4% paraformaldehyde for 15 minutes. After twice washing with PBS, the fixed cells were permeabilized in PBS containing 0.5% Triton X‐100 at 37°C for 90 minutes. Thereafter, TUNEL staining was performed for each sample in the dark at 37°C. Following 3 × PBS wash, cells were incubated with 100 μL of DAPI (1 μg/mL) to stain nuclei and photographed by using a fluorescent microscope. The apoptotic index was expressed as the percentage of apoptotic cells (TUNEL‐positive) compared with total cells (DAPI‐positive). Five randomly selected microscopic fields in each group were used to calculate the apoptotic index, and all the experiments were performed three times.

### 2.7 Flow cytometry

The assessment of apoptosis was performed by employing the Annexin V–FITC Apoptosis Detection Kit according to the manufacturer's protocol as described previously.^15^ Briefly, after treatment, the cells were harvested, washed twice with ice‐cold PBS, suspended in 500 μL of 1 × binding buffer and then double‐stained with Annexin V‐FITC and PI each in 5 μL for 5 minutes in the dark at room temperature. Finally, the sample was analysed with FACSCalibur flow cytometer (Becton Dickinson, Mansfield, MA, USA) and CellQuest software (BD Biosciences) were used to analyse the apoptotic cells.

### 2.8 Western blot assay

After treatment, the harvested cells were washed twice with ice‐cold PBS and lysed in RIPA lysis buffer. The lysates were centrifuged at 13 000 rpm for 15 minutes at 4°C, and the resulting supernatants were collected to determine the protein content with a BCA protein assay kit according to the manufacturer's instructions. Each sample with 20 μg of total protein was decentralized by 12% sodium dodecyl sulphate polyacrylamide gel electrophoresis (SDS‐PAGE) and electrophoretically transferred to nitrocellulose membranes. 5% non‐fat milk was added to block the membrane for 1 hour at room temperature and then incubated overnight with the primary antibody (Bax, Bcl‐2 and cleaved caspase‐3) at a dilution of 1:1,000 at 4°C. After being rinsed with PBS for three times, the samples were incubated with appropriate horseradish peroxidase‐conjugated secondary antibody (1:5000) at room temperature for 1 hour. The protein bands image was developed using an enhanced chemiluminescence (ECL) reagents, and band intensities were quantified with ImageQuant LAS 4000 (Pittsburgh, PA, USA). β‐actin was used as an internal control. All experiments were repeated three times.

### 2.9 Measurement of intracellular ROS

The level of intracellular ROS formation was determined using 2,7‐dichlorofluorescein diacetate (DCFH‐DA) as previously described.^7^ Briefly, treated cells were stained with 10μM DCFH‐DA at 37°C in the dark for 30 minutes followed by twice PBS wishing, and immediately imaged on a fluorescence microscope before being subjected to flow cytometric examination (BD FACSCalibur, Becton Dickinson, Mansfield, MA, USA) using the CellQuest software (BD Biosciences, Becton Dickinson). All values of ROS level were normalized to the control group and expressed as a percentage of the control group (100%).

### 2.10 Antioxidant assay

After treating cells in different conditions, total cell extracts were prepared and protein concentration was determined by Bradford method as described by Yu et al^16^ The activities of SOD, CAT and GPX, and the content of MDA and GSH were measured spectrophotometrically using respective commercial diagnostic kits. All experimental procedures are strictly followed by the manufacturer's protocols. For all antioxidant enzymes (SOD, CAT, GPX), the activity is expressed as units per mg protein. Lipid peroxidation marker MDA and glutathione (GSH) were expressed as nmol of per gram protein.

### 2.11 Statistical analysis

All data were expressed as means ± standard deviation (SD). All statistical analyses were conducted using GraphPad Prism software version 5.0. The statistical differences were carried out using one‐way ANOVA followed by Tukey's multiple comparison. A value of *P* < .05 were regarded statistically significant. All experiments were performed at least in triplicates.

## 3 RESULTS

### 3.1 FA prevented H_2_O_2_‐induced cell damage and death in ARPE‐19 cells

**Figure 1:**
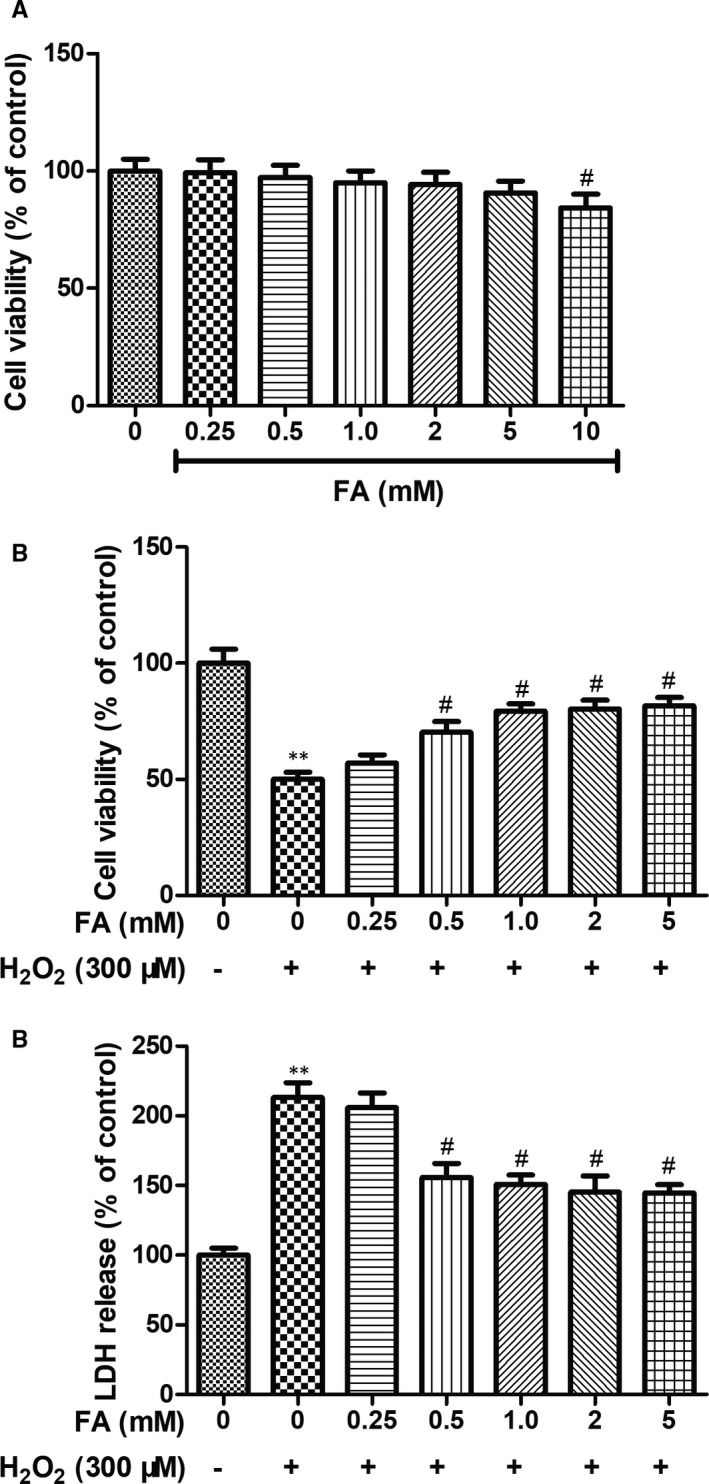
Protective effects of FA against H_2_O_2_‐induced cytotoxicity in ARPE‐19 cells. A, The effect of FA on the cell viability of ARPE‐19 cells detected by MTT assay; ARPE‐19 cells were pre‐treated with different concentration of FA (0, 0.25, 0.5, 1, 2, 5 and 10 mM) for 24 h. B, The effect of FA pre‐treatment on the cell viability of H_2_O_2_‐induced human ARPE‐19 cells detected by MTT assay; ARPE‐19 cells were pre‐treated with different concentration of FA (0, 0.25, 0.5, 1, 2 and 5mM) for 24 h and then treated with H_2_O_2_ (300 μM) for 4 h. C, The effect of FA pre‐treatment on the cell death of H_2_O_2_‐induced human ARPE‐19 cells detected by LDH assay. ARPE‐19 cells were pre‐treated with different concentration of FA (0, 0.25, 0.5, 1, 2 and 5mM) for 24 h and then treated with H_2_O_2_ (300 μM) for 4 h. Each column presented means ± SD (n = 3). ^**^ *P* < .01 vs normal control group. ^#^ *P* < .05, ^##^ *P* < .01 vs H_2_O_2_‐treated group

**Figure 2:**
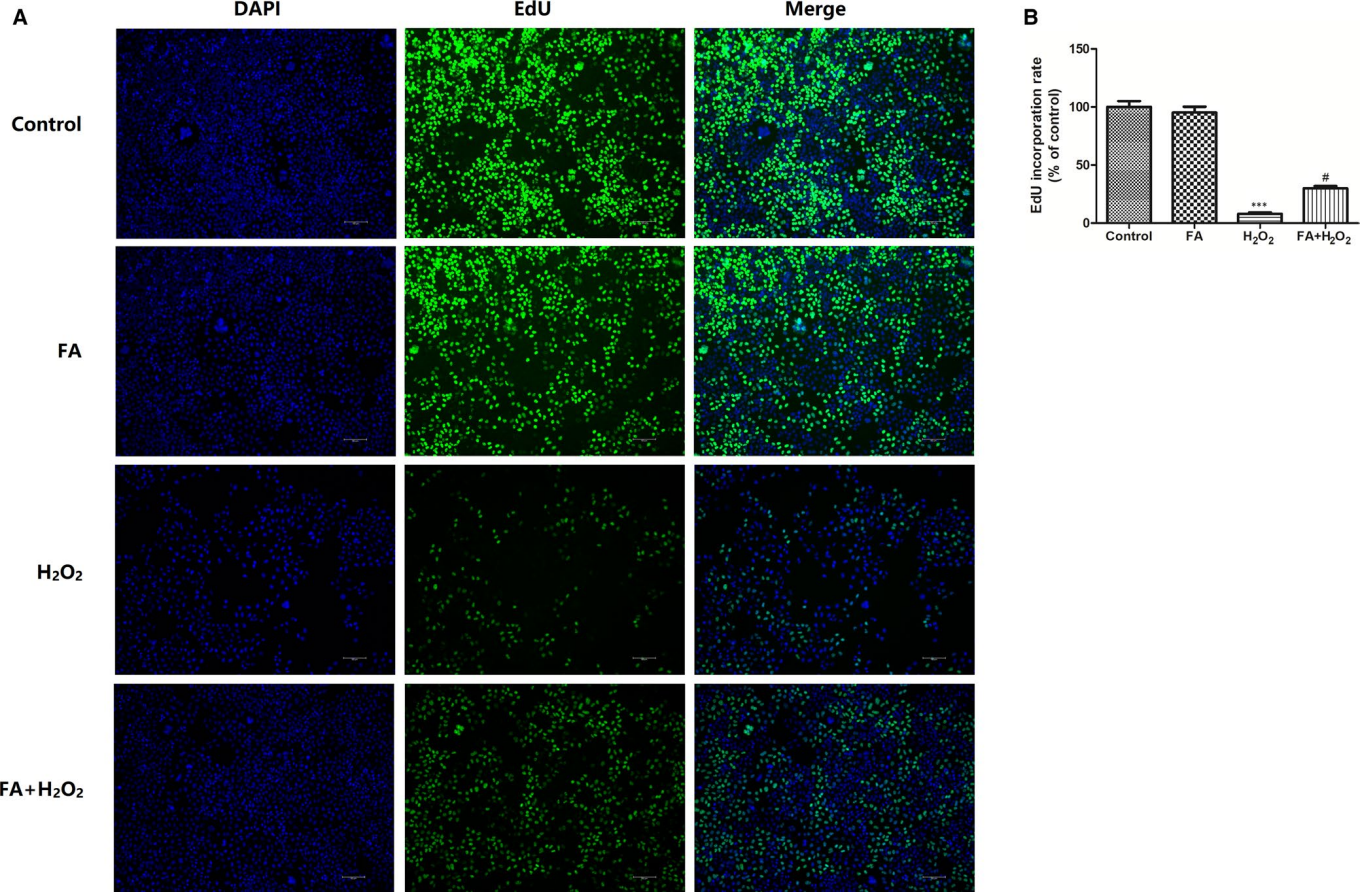
Protective effects of FA against H_2_O_2_‐induced cell loss in ARPE‐19 cells. A, The effect of FA pre‐treatment on the proliferation of H_2_O_2_‐induced human ARPE‐19 cells detected by EdU incorporation assay; (B) Quantitative results of the EdU incorporation assay. ARPE‐19 cells were pre‐treated with FA (5 mM) for 24 h and then treated with H_2_O_2_ (300 μM) for 4 h. Each column presented means ± SD (n = 3). ^***^ *P* < .001 vs normal control group. ^#^ *P* < .05 vs H_2_O_2_‐treated group

The cytotoxicity of FA on human ARPE‐19 cells was first examined after 24 hours treatment. As shown in Figure 1A, cell viability kept unchanged until the high concentration of 5 mM in ARPE‐19 cells treated with FA compared to the control group (*P* > .05), whereas beyond this concentration, less cell loss was obviously observed in cells treated with FA at 10 mM compared to the control group (*P* < .05). Next, to investigate whether FA can protect RPE cells from H_2_O_2_‐induced cell death, ARPE‐19 cells were incubated with FA for 24 hours before being replaced with a fresh culture medium containing H_2_O_2_ (300 μM) for 4 hours. As demonstrated in Figure 1B, challenge with 300 μM H_2_O_2_ for 4 hours led to a significant cell loss of 55%. On the contrary, in the ageing state, FA pre‐treatment for 24 hours reversed this trend in a dose‐dependent manner especially beyond 0.25 mM when compared with H_2_O_2_ treated control (*P* < .05). Furthermore, H_2_O_2_‐induced LDH leakage was also dramatically reduced in cells pre‐treated with the same concentrations of FA (Figure 1C). In line with this observing, EdU incorporation assay (Figure 2A,B) disclosed that H_2_O_2_ challenge resulted in marked less cell proliferation (lower green fluorescence intensity) of ARPE‐19 cells when compared with normal control (*P* < .001), whereas more cell proliferation (higher green fluorescence intensity) was observed in ARPE‐19 cells in response to FA (5 mM) pre‐treatment.

### 3.2 FA attenuated H_2_O_2_‐induced apoptosis in ARPE‐19 cells

**Figure 3:**
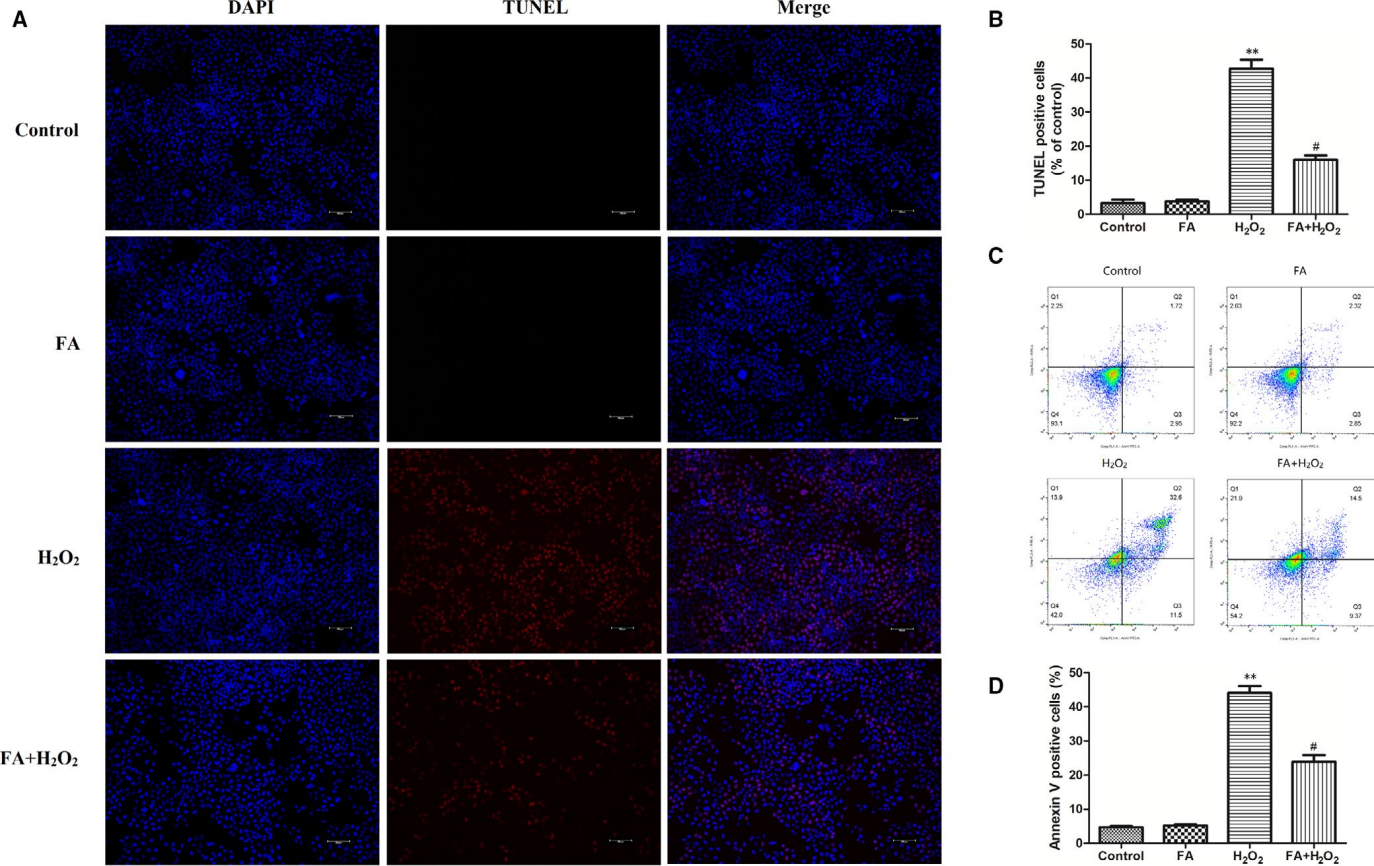
Protective effects of FA against H_2_O_2_‐induced cell apoptosis in ARPE‐19 cells. A, The effect of FA pre‐treatment on the apoptosis of H_2_O_2_‐induced human ARPE‐19 cells detected by TUNEL assay; (B) Quantitative results of the TUNEL assay; (C) Flow cytometry analysis of cell apoptosis using Annexin V‐FITC/PI dual‐staining; (D) Quantitative results of the rate of apoptotic cells. Annexin V‐positive cells (Q2 + Q3) was calculated for each group cells and are shown in the bar graph. ARPE‐19 cells were pre‐treated with FA (5 mM) for 24 h and then treated with H_2_O_2_ (300 μM) for 4 h. Each column presented means ± SD (n = 3). ^**^ *P* < .01 vs normal control group. ^#^ *P* < .05 vs H_2_O_2_‐treated group

Next, in order to evaluate if this protective effect of FA on H_2_O_2_‐induced cell death was related to apoptosis, ARPE‐19 cells pre‐treated with or without FA (5 mM) for 24 hours were further exposed to 300 μM H_2_O_2_ for 4 hours, and then subject to TUNEL staining assay. As shown in Figure 3A,B, the addition of FA (5 mM) alone showed no apoptosis induction effect on ARPE‐19 cells (*P* > .05), but 4‐h stimulation of 300 μM H_2_O_2_ led to a significant increase of apoptotic cells than that of the control (*P* < .01), as showed by the increased percentage of TUNEL‐positive cells in DAPI‐positive cells. Interestingly, pre‐incubation with FA for 24 hours significantly alleviated the apoptosis induced by 4‐hour challenge of H_2_O_2_, with 16.27% apoptotic cells when compared with the cells treated with H_2_O_2_ alone (42.70%, *P* < .05). Under the same condition, AV‐FITC and PI staining of ARPE‐19 cells were also performed on flow cytometry to examine the status of apoptosis. In consistency with the above result, H_2_O_2_ stimulation for 4h evoked a high proportion of apoptotic cells in ARPE‐19 cells when compared with the untreated control (*P* < .01, Figure 3C,D), while pre‐treatment with FA directly decreased apoptosis rate induced by H_2_O_2_.

### 3.3 FA regulated H_2_O_2_‐induced apoptosis‐related protein expression in ARPE‐19 cells

**Figure 4:**
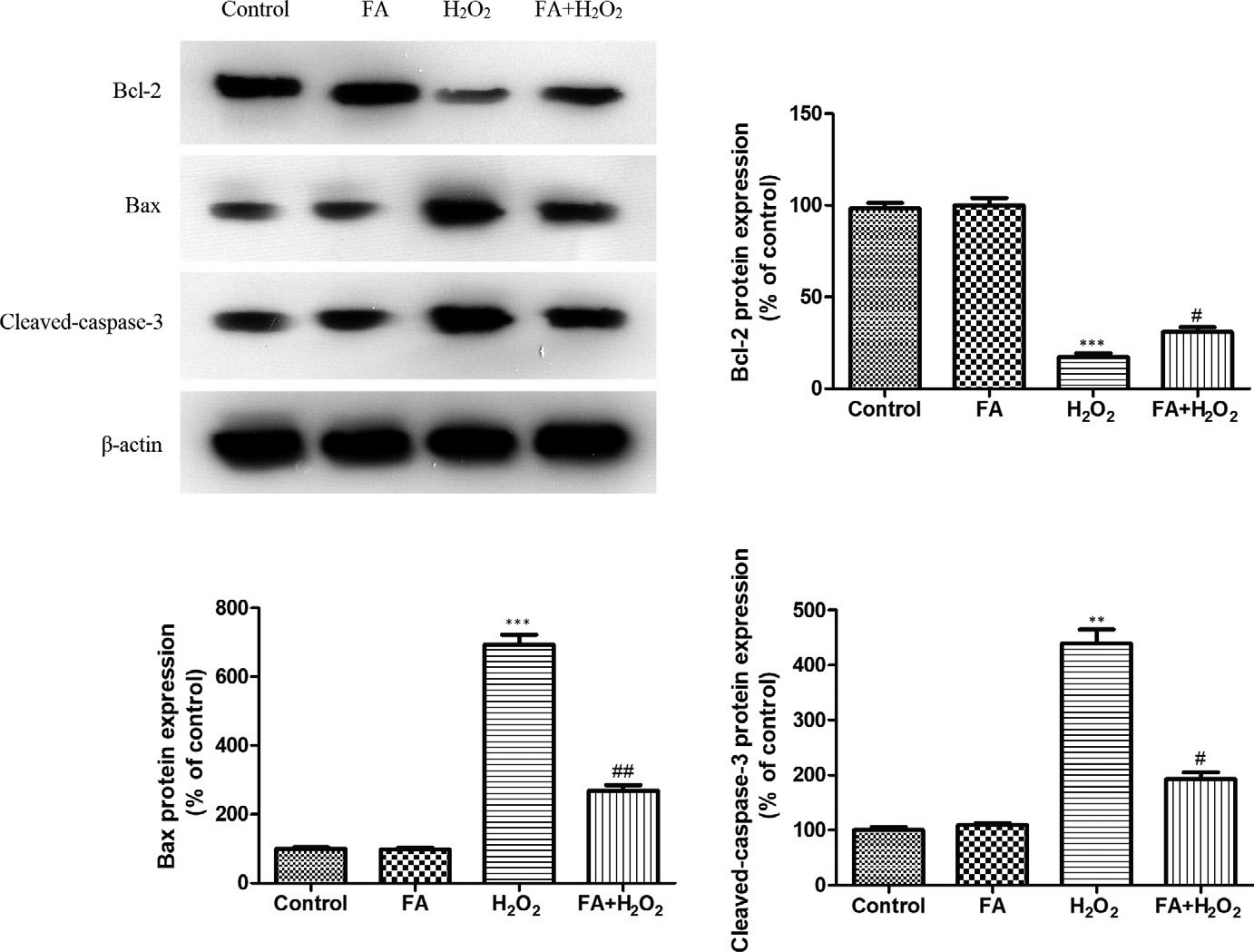
The effect of FA pre‐treatment on H_2_O_2_‐induced change of Bax, Bcl‐2 and cleaved caspase‐3 protein expression in ARPE‐19 cells. ARPE‐19 cells were pre‐treated with FA (5 mM) for 24 h and then treated with H_2_O_2_ (300 μM) for 4 h. Each column presented means ± SD (n = 3). ^**^ *P* < .01, ^***^ *P* < .001 vs normal control group. ^#^ *P* < .05, ^##^ *P* < .01 vs H_2_O_2_‐treated group

Considering the protective effect of FA against H_2_O_2_‐induced apoptosis, we detected the expression change of Bax, Bcl‐2 and cleaved caspase‐3 in ARPE‐19 cells by Western blotting under same conditions. Figure 4 showed that pre‐treatment with FA (5 mM) alone did not cause any change of these three proteins, while a significant increased Bax and caspase‐3 protein expression occurred in ARPE‐19 cells challenged with 300 μM H_2_O_2_, in combination with a decreased Bcl‐2 protein expression. Pre‐treatment FA for 24 hours intensely down‐regulated Bax and cleaved caspase‐3 protein expression, but up‐regulated the Bcl‐2 protein expressing as compared to those stimulated with H_2_O_2_ alone (*P* < .05 or *P* < .01).

### 3.4 FA ameliorated H_2_O_2_‐induced oxidative stress and lipid peroxidation in ARPE‐19 cells

**Figure 5:**
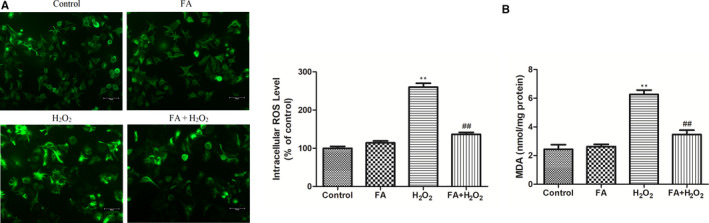
The effect of FA pre‐treatment on H_2_O_2_‐induced oxidative stress and lipid peroxidation in ARPE‐19 cells. A, The effect of FA pre‐treatment on H_2_O_2_‐induced change of ROS in ARPE‐19 cells; (B) The effect of FA pre‐treatment on H_2_O_2_‐induced change of MDA in ARPE‐19 cells. ARPE‐19 cells were pre‐treated with FA (5 mM) for 24 h and then treated with H_2_O_2_ (300 μM) for 4 h. Each column presented means ± SD (n = 3). ^**^ *P* < .01 vs normal control group. ^##^ *P* < .01 vs H_2_O_2_‐treated group

The ROS secretion in different group was measured by DCFH‐DA staining (Figure 5A). Compared with the control cells, FA alone treatment did not cause any ROS change. Exposure of ARPE‐19 cells to H_2_O_2_ lead to a 2.7‐fold increase of ROS generation compared with control, but pre‐treatment of cells with FA lowered down this increase to 1.5‐fold of control cells. As for MDA, FA (5 mM) showed the same tendency as observed for ROS in ARPE‐19 cells (Figure 5B). Increased MDA level by H_2_O_2_ was significantly mitigated in ARPE‐19 cells pre‐treated with FA for 24h. Similarly, no change of MDA was observed in FA alone treated group compared with control (*P* > .05).

### 3.5 FA improved antioxidant enzymes activities and glutathione content in ARPE‐19 cells

**Figure 6:**
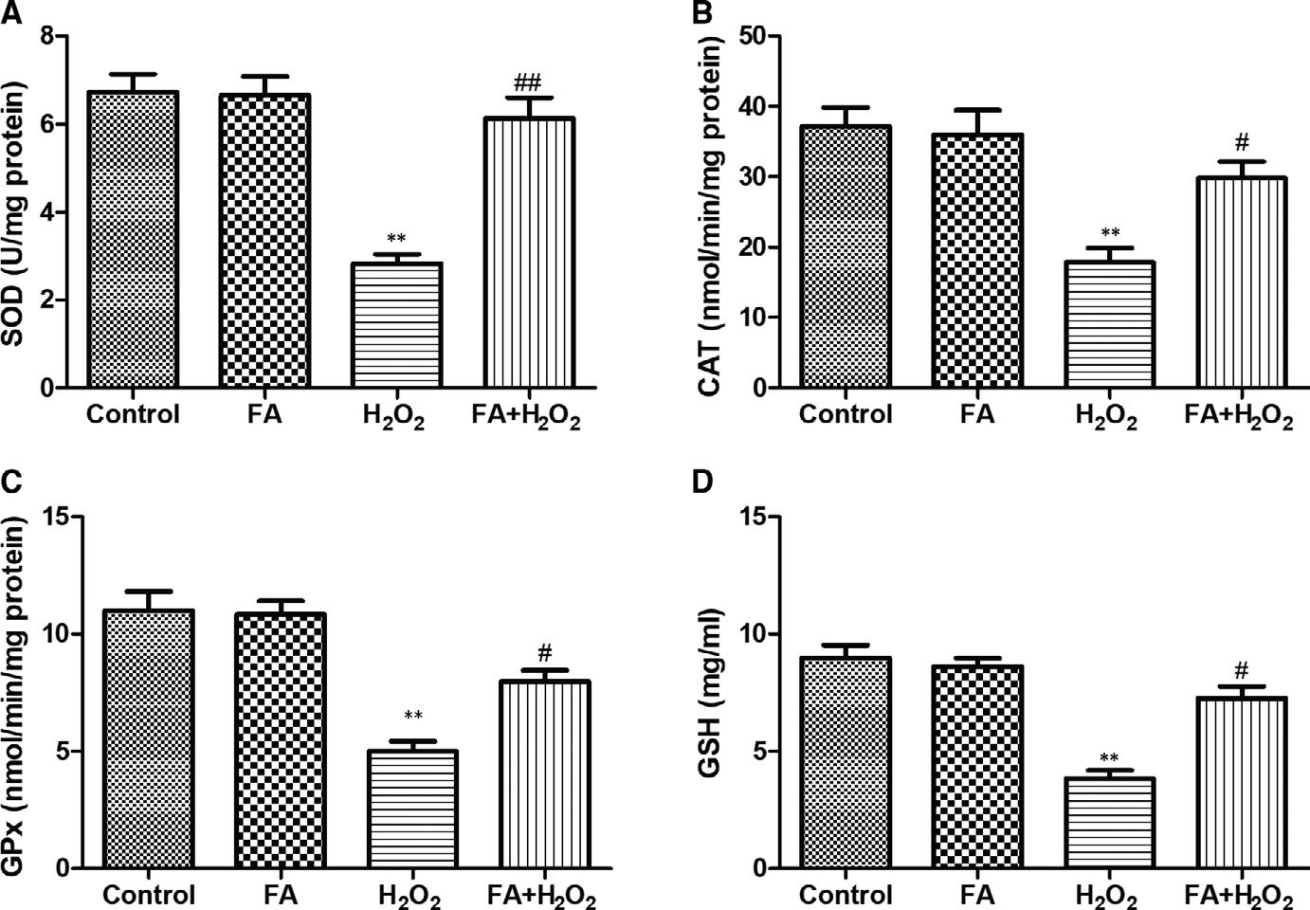
The effect of FA pre‐treatment on antioxidant status in ARPE‐19 cells. A, The effect of FA pre‐treatment on SOD activity in ARPE‐19 cells; (B) The effect of FA pre‐treatment on CAT activity in ARPE‐19 cells; (C) The effect of FA pre‐treatment on GSH‐PX activity in ARPE‐19 cells; (D) The effect of FA pre‐treatment on GSH level in ARPE‐19 cells. ARPE‐19 cells were pre‐treated with FA (5 mM) for 24 h and then treated with H_2_O_2_ (300 μM) for 4 h. Each column presented means ± SD (n = 3). ^**^ *P* < .01 vs normal control group. ^#^ *P* < .05, ^##^ *P* < .01 vs H_2_O_2_‐treated group

As shown in Figure 6, exposure of ARPE‐19 cells to 300μM H_2_O_2_ resulted in a significant decline of SOD, CAT and GSH‐PX activities, as well as GSH in ARPE‐19 cells when compared with untreated control (*P* < .01). When ARPE‐19 cells were pre‐cultured with FA (5 mM) for 24 hours and then co‐incubated with H_2_O_2_ (300 μM) for another 4 hours, SOD, CAT, and GSH‐PX and GSH levels were increased in different scale to approach the normal level, which were all statistically different from H_2_O_2_ group (*P* < .05 or *P* < .01). In addition, no change of these oxidative stress biomarkers was observed in cells treated with FA alone.

## 4 DISCUSSION AND CONCLUSIONS

The RPE is a single layer of pigmented cells locating adjacent to the outer retina layer, where it forms partial blood‐retina barrier and performs functions that are essential for maintaining the structural integrity of the retina.^17^, ^18^ This process involves transportation of oxygen and nutrients, uptake of circulating vitamin A, elimination of wastes of photoreceptors metabolism and secretion of essential factors for facilitating the regeneration and repair of retina.^19^ It is found that the retina contains highest oxygen consumption than other tissues, which suggests that RPE cells are susceptible to oxidative stress, particularly when exposed to high levels of reactive oxygen species (ROS).^20^ Furthermore, RPE is lack of ability to renew itself following differentiation ^7^ and the resistance of the antioxidant system of RRE decreases in the elderly population.^21^ In this regard, oxidative stress and declined antioxidant capacity result in functional disorders and structural abnormalities of the RPE, such as lipid peroxidation, oxidation of enzymes, and mitochondrial DNA breakage, which have been regarded as important pathological alterations associated with AMD.^22^ Accordingly, the extensive degeneration of RPE induced by ROS may cause the death of retina photoreceptor cells, thereby leading to retinal degeneration and even irreversible vision loss in AMD patients with increasing age.^23^, ^24^ In addition, previous studies have suggested that ROS‐induced cumulative damage to RPE cells is the key event in the early stage of AMD.^25^ As such, early interventions targeting at rescuing RPE cells from oxidative stress‑induced damage might be a possible good strategy to prevent or delay the progression of AMD.

As we know, H_2_O_2_ is a principal factor inducing oxidative damage and executing cell death in many types of cells, including retinal cells.^26^ Here, in this study, an in vitro ageing model established on ARPE‐19 cells with H_2_O_2_ challenge for 4h was explored to mimic the pathogenesis of AMD. MTT showed that FA at the concentration ranging from 0.25‐5 mM exhibited not any cytotoxicity on human ARPE‐19 cells, indicating the safe concentration of FA on ARPE‐19 cells. As expected, nearly half of cell loss was observed in H_2_O_2_ exposed cells, as evidenced by the reduced percentage of cell viability by MTT assay. Similarly, the cellular release of LDH, serving as a signal of cellular damage/integrity, was also increased in H_2_O_2_ exposed cells, as determined by LDH assay. In contrast, pre‐treatment of ARPE‐19 cells with increasing concentration of FA (5 mM) for 24 hours prior to H_2_O_2_ exposure significantly increased cell viability. EdU incorporation staining assay further demonstrated that FA made great contribution to restore or enhance the cell proliferation of ARPE‐19 cells under H_2_O_2_ stimulated condition. These findings suggested that FA possessed a protective effect against H_2_O_2_‑induced oxidative damage in RPE cells by improving cell proliferation.

There is abundant evidence suggesting that oxidative stress‐induced apoptosis in RPE cells is the leading cause of AMD.^27^ Considering this increased cell viability of H_2_O_2_ challenged‐ARPE‐19 cells in the presence of FA, we next examine if this effect was achieved by inhibition of apoptosis induced by H_2_O_2_. TUNEL staining assay indicated that pre‐treatment with FA (5 mM) for 24 hours can dramatically suppress the percentage of apoptotic ARPE‐19 cells induced by H_2_O_2,_ as showed by a significant decrease of the percentages of purplish red‐stained TUNEL‐positive cells. In line with this observing, flow cytometry disclosed that the increased apoptosis rate induced by H_2_O_2_ was definitely inhibited in ARPE‐19 cells pre‐treatment with FA for 24h. In these assays, FA pre‐treatment alone did affect the cell growth and apoptosis of ARPE‐19 cells. This result clearly demonstrated that FA can effectively protect ARPE‐19 cells from H_2_O_2_‐induced cell death via inhibition of apoptosis.

To make an in‐depth investigation for this anti‐apoptotic ability of FA against H_2_O_2_, apoptosis‐related proteins including Bax, Bcl‐2 and cleaved caspase‐3 were assessed in ARPE‐19 cells. Western blot result revealed that FA itself exerted no effect on the change of these proteins, but the addition of H_2_O_2_ resulted a marked increase of Bax and cleaved caspase‐3 protein expression in combination with a decreased Bcl‐2 protein expression. Of note, a 24 hours of FA pre‐treatment caused this change to the opposite, suggesting the protective effect of FA on H_2_O_2_‐induced cell death was mediated by regulation of Bax, Bcl‐2 and cleaved caspase‐3 protein expression.

Recent studies have suggested that H_2_O_2_‐induced apoptosis was believed to be related to increased levels of ROS in the mitochondria of RPE cells and this over‐production of ROS can be reversed by enhancing antioxidant enzymes, thus in turn alleviating apoptotic status of aged RPE cells.^28^, ^29^ As such, DCFH‐DA staining was first used to determine the ROS production in ARPE‐19 cells following different treatment. The fluorescence intensity reflected the amount of ROS, namely the more ROS remains, and vice versa.^6^ Quantitative fluorescence intensities analysis indicated that, in the absence of FA, the fluorescence intensity of ROS was higher than control cells. On the contrary, addition of FA demolished the DCF fluorescence triggered by H_2_O_2_, closed to the extent of control cells. In addition, MDA level, an indicator of lipid peroxidation, was present in the same manner. Due to the vital role of oxidative stress in the ageing status of RPE cells, we measured the activities of SOD, CAT, and GSH‐PX, and GSH level in ARPE‐19 cells following different treatment. Pre‐treatment with FA can dramatically alleviate the decline of these four parameters induced by H_2_O_2_, although FA itself had not any effect on their level change. These results suggested FA attenuated H_2_O_2_‐induced intracellular ROS production in ARPE‐19 cells via improvement of endogenous anti‐oxidative activity.

In summary, the present findings demonstrated for the first time that FA was able to effectively protect H_2_O_2_‐induced cell damage via suppression of apoptosis, promotion of cell proliferation and activation of anti‐oxidative activity, which shed light on the potential use of FA for the prevention or treatment of AMD.

## CONFLICT OF INTEREST

The authors have no commercial or other associations that might pose a conflict of interest.

## AUTHOR CONTRIBUTIONS

**Kunpeng Xie:** Data curation (equal); Formal analysis (equal); Investigation (equal); Methodology (equal); Writing‐original draft (equal); Writing‐review & editing (equal). **Bo Jin:** Methodology (equal); Software (equal); Validation (equal); Visualization (equal). **Haiyan Zhu:** Investigation (equal); Methodology (equal); Resources (equal); Software (equal). **Pengyi Zhou:** Data curation (equal); Formal analysis (equal); Investigation (equal); Validation (equal); Visualization (equal). **Liping Du:** Methodology (equal); Validation (equal); Visualization (equal). **Xuemin Jin:** Conceptualization (equal); Data curation (equal); Investigation (equal); Project administration (equal); Supervision (equal); Writing‐original draft (equal); Writing‐review & editing (equal).

## Data Availability

All data generated or analysed during this study are included in this article.
